# 3D Hermite Transform Optical Flow Estimation in Left Ventricle CT Sequences

**DOI:** 10.3390/s20030595

**Published:** 2020-01-21

**Authors:** Carlos Mira, Ernesto Moya-Albor, Boris Escalante-Ramírez, Jimena Olveres, Jorge Brieva, Enrique Vallejo

**Affiliations:** 1 Facultad de Ingeniería, Universidad Nacional Autónoma de México, Ciudad de México 04510, Mexico; jolveres@gmail.com; 2 Facultad de Ingeniería, Universidad Panamericana, Augusto Rodin 498, Ciudad de Mexico 03920, Mexico; emoya@up.edu.mx (E.M.-A.); jbrieva@up.edu.mx (J.B.); 3 Centro Médico ABC, Ciudad de México 01120, Mexico; vallejo.enrique@gmail.com

**Keywords:** bio-inspired computing, motion estimation, optical flow, differential method, steered hermite transform, cardiac CT imaging, algorithms

## Abstract

Heart diseases are the most important causes of death in the world and over the years, the study of cardiac movement has been carried out mainly in two dimensions, however, it is important to consider that the deformations due to the movement of the heart occur in a three-dimensional space. The 3D+t analysis allows to describe most of the motions of the heart, for example, the twisting motion that takes place on every beat cycle that allows us identifying abnormalities of the heart walls. Therefore, it is necessary to develop algorithms that help specialists understand the cardiac movement. In this work, we developed a new approach to determine the cardiac movement in three dimensions using a differential optical flow approach in which we use the steered Hermite transform (SHT) which allows us to decompose cardiac volumes taking advantage of it as a model of the human vision system (HVS). Our proposal was tested in complete cardiac computed tomography (CT) volumes ( 3D+t), as well as its respective left ventricular segmentation. The robustness to noise was tested with good results. The evaluation of the results was carried out through errors in forwarding reconstruction, from the volume at time *t* to time t+1 using the optical flow obtained (interpolation errors). The parameters were tuned extensively. In the case of the 2D algorithm, the interpolation errors and normalized interpolation errors are very close and below the values reported in ground truth flows. In the case of the 3D algorithm, the results were compared with another similar method in 3D and the interpolation errors remained below 0.1. These results of interpolation errors for complete cardiac volumes and the left ventricle are shown graphically for clarity. Finally, a series of graphs are observed where the characteristic of contraction and dilation of the left ventricle is evident through the representation of the 3D optical flow.

## 1. Introduction

Cardiovascular diseases (CVDs) take the lives of 17.9 million people every year, 31% of all global deaths, this represents the number one cause of death globally, more people die annually from CVDs than from any other cause [1]. Heart diseases as myocardial infarction, ischemia or hypertrophy can be characterized by analyzing the dynamics of the heart. During the cardiac cycle (contraction “systole” and relaxation “diastole” of the heart), the motion wall estimation can be used to recognize those pathologies. The acquisition of cardiac volumes has allowed quantifying relevant left ventricular (LV) parameters such as its volume, strain, twist, and desynchrony [2]. Nowadays, there are diagnostic imaging techniques to characterize cardiac anatomy and function, as such echocardiography, cardiac Magnetic Resonance Imaging (MRI), cardiac Computed Tomography (CT), cardiac Positron Emission Tomography (PET), and coronary angiography [3]. Where the cardiac CT technique has certain advantages with respect other: a higher resolution that ultrasound, it is more accessible than MRI and it is a non-invasive a fast imaging option [4]. Cardiac CT allows acquiring three-dimensional morphological images, with motion artifacts minimizes, and showing the heart chambers and the coronary arteries at different planes [5]. It is then possible to acquire good quality cardiac CT data of the heartbeats. Images are acquired overall cardiac cycles to produce the final volume image. Due to the complexity of the heart motion, it is still hard for the physician to estimate the 3D motion during the exam, thus, it is necessary to develop computational analysis tools to aid in the diagnosis process.

The human heart is a complex organ in terms of anatomy and physiology, the estimation of its movement is an important task to understand its mechanism and to assist in the medical diagnosis. Different image processing techniques can be applied to calculate and to observe the motion of the heart, for example, the optical flow estimation is a method used in those situations where the correspondence between the pixels, within an image sequence, is required. The optical flow methods compute an approximation to the 2D motion in an image sequence from spatiotemporal patterns of image intensity [6]. Over the years, state-of-the-art algorithms for optical flow can be summarized in Nagel [7] who identified the common rigorous restrictions [8,9,10], as well as smoothing restrictions for the optical flow solution [7,11,12]. On the other hand, Barron et al. [6], categorized the optical flow in four groups: differential techniques [7,8,9,11,13,14], region-based matching [15,16], energy-based methods [17,18] and phase-based techniques [19]. Sun et al. [20] suggested that there have been few changes in the typical formulation given by Horn and Schunck [11]. Most of research work on optical flow has been carried out in 2D+t. Many of them claim to be 3D, but they really are 2D+t. Some of them, use 2D projections to obtain a 3D representation, in applications such as tracking traffic [21], in methods used for quantitative motion estimation of biological structures in light microscope [22], estimation of 3D geometry and 3D motion using spatiotemporal gradients [23] or emotion recognition from 3D videos [24]. 2D optical flow estimation has been used in the heart analysis to identify patients with some diseases, recent works using 2D optical flow cover topics such as motion estimation in cardiac fluorescence imaging [25], and as the automatic localization of the heart from cine MRI [26]. Some optical flow methods have used image models inspired by nature, for example Gabor filters [18,19,27,28,29,30]. The optical flow is fundamentally different than tracking because a complete set of correspondences between the pixel levels in an image (or volume) is obtained. The optical flow is used to calculate dense trajectories, provides more freedom and information about the data in which the movement is being estimated, a priori models are not needed and even more, it can also be used to develop deformable based-model tracking algorithms.

The Hermite transform (HT) has been an image model used to describe the local constraints of the Horn and Schunck approach. Liu et al. [27] derived a six-parameter non-affine optical flow model, which is solved with high-order Hermite polynomial filtered data. In [28], Silvan et al. showed that through a linear mapping of 3D Hermite coefficients by specific projection functions, we could obtain the Hermite transform coefficients of local projections. Furthermore, Moya et al. [29] used the steered Hermite coefficients like local motion restrictions, found in current methods, to define a differential estimation method.

One disadvantage of the 2D cardiac movement analysis is that it is constrained by geometry-dependent reference directions of deformation (i.e., radial, circumferential, and longitudinal). In this sense, a 3D cardiac movement analysis may overcome such limitations by referencing the intrinsic directions of deformation [31]. Thus, to identify altered ventricular function in patients with CVD, a 3D left ventricular (LV) deformation analysis is more suitable since it represents contributions from counter-directional, helically arranged fibers shortening and thickening throughout the cardiac cycle [32]. Research on the measurement of cardiac motion has been commonly made in 2D+t [33] but this analysis should be done in 3D+t to enable us to describe the true motions of the heart, for example, the twisting motion that takes place on every beat cycle. Compared to the 2D analysis, the 3D analysis has not received much attention, although there are currently working groups analyzing optical flow in 3D mainly using ultrasound images.

About the estimation of 3D optical flow in general, some works have similarities with at least one of the aspects of this article, thereby, in [34] they use a 3D model of the human body and motion captured data to synthesize flow fields and train a convolutional neural network (CNN) to estimate human flow fields from pairs of images. In [35] a steerable filter-based algorithm is formulated, in its simplest form, for estimating 3D flow in sequences of volumetric or point-cloud data.

In [36] they present an approach for real-time respiratory motion estimation in image-guided interventions by employing contrast-invariant feature descriptors. Yoon et al. [37] presented a method for motion estimation applied to cone-beam CT, their work uses an energy functional, which includes as terms: a data fidelity, a regularization term, and the optical flow restriction. On the other hand, Jungwon et al. [38] used the optical flow estimation to calculate the local motion, allowing a 3D segmentation extension. Their model includes a shape distortion over time term, allowing segmenting and tracking the lung nodules. In [39], an implementation based on the optical flow algorithm from Farnebäck (2003) is used to create 3D freehand ultrasound but with reconstructions from 2D without external tracking, using deep learning.

Several methods have been used to estimate the optical flow of the endocardial wall motion [40]. In [41], a global anatomically constrained affine optical flow tracking was used to track the end-diastole left ventricle surface throughout the cardiac cycle. For [42], this approach first performs 3D segmentation at the end-diastolic frame and then performs tracking over the cardiac cycle using both global (optical flow) and local (block matching) methods. In [43] they claim to have a method for detecting cardiac flow in echocardiography where the sampling planes representing the mitral inflow tract and the left ventricle outflow tract are traced by fusing information from multiple cues, including optical flow, boundary detection, and motion prior. Duan et al. [44], evaluate a correlation-based optical flow algorithm for tracking endocardial surfaces on three-dimensional ultrasound data, also in [45] they built a truly 3D mathematical phantom of cardiac tissue and blood in order to validate the optical flow for quantification of myocardial deformations. Leung et al. [46] track left ventricular borders in 3D echocardiographic sequences by combining differential optical flow with statistical modeling. Zhiang et al. [47] developed an optical flow algorithm based on Thirion’s diffusing model [48], also known as the ‘‘demons’’ algorithm and also described an atlas-based geometry pipeline for constructing three-dimensional cubic Hermite finite element meshes of the human heart.

In Table 1 we summarized some of the most recent optical flow motion methods used to extract the motion estimation, either using a 2D+t or 3D+t model, and the differences with the proposed method. In some cases, a 2D optical flow is initially estimated to map it onto a 3D optical flow.

The present article is an extension of our previous work published in [49]. In that work, we proposed the three-dimensional optical flow estimation using the 3D steered Hermite transform and we compared our approach with the 3D Horn-Schunck method. In contrast, the current work is compared with a multiresolution Horn and Schunck approach reported by Sun et al. [20], moreover, in this work, we perform a depth analysis about the optimal parameters of the method proposed and we focused the Section 5.2.1 to analyzed the 3D optical flow estimation of the left ventricle, first, showing its 3D segmentation and showing the advantages of our approach compared with the 3D version of the method of Sun et al. [20], and then, showing the 3D motion of the left ventricle in different cardiac cycle and a whole cardiac cycle, highlighting the corresponding contraction and relaxation movements present in each phase of the cardiac cycle. It should be mentioned that similar 2D algorithms using the Hermite transform have already been presented in [29,33] but with the main disadvantage of consuming a lot of computing time.

Revising the algorithm from 2D to 3D is not a trivial problem, on top of the additional and necessary computational complexity, and the importance of describing the 3D cardiac movement. We have to describe (based on [50]) and calculate a second local orientation angle from the 3D cartesian coefficients of Hermite to obtain the 3D steered coefficients of Hermite (SHT3D). The data used in this work require a sensitivity analysis of its parameters and a way to validate the results because there aren’t annotated volumes. Robust noise tests and calculation of interpolation errors of the volumes used had been carried out. Left ventricular analysis has been of great importance for this article. In this way, the results of the optical flow and the segmentation of such cardiac structure were evaluated.

**Figure 1:**
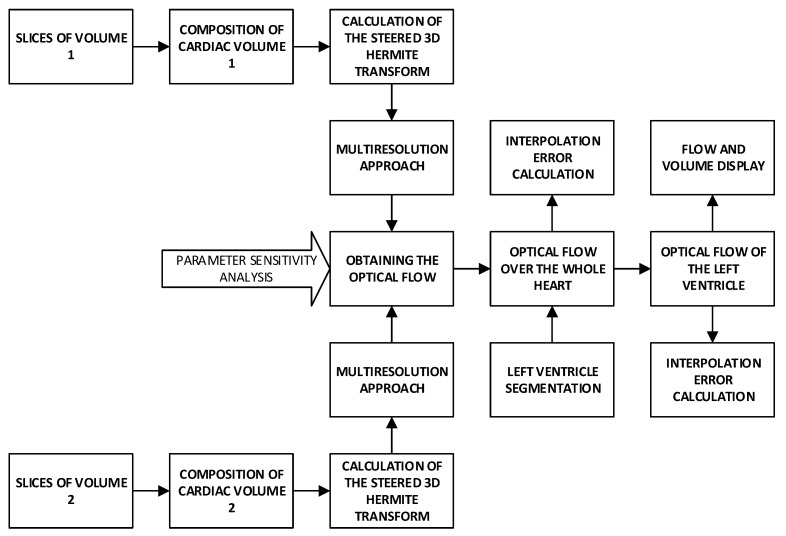
Overview of the proposed method.

Figure 1 shows an overview of the proposed method according to the procedures explained in the next sections.

Our approach uses a three-dimensional (3D+t), that is, it uses the data of the cardiac volumes in a three-dimensional space (x,y,z), which change over time during the entire cardiac cycle; a modified version of Sun et al. [20], which, in contradistinction of Horn and Schunck’s approach [11], uses an incremental multiresolution technique to estimate large displacements, where the optical flow at a coarse level is extrapolated to warp the second image at a finer level, combined with the optical flow based on the Hermite transform proposed by Moya et al. [29], that uses the several constraints found in the more accurate optical flow methods. The rest of the paper is organized as follows: Section 2 describes the 3D Hermite transform, Section 3 develops the proposal to obtain 3D optical flow, Section 5 presents the experimental results and discussion of this work, Section 6 is about the results obtained, and finally, Section 7 concludes the paper and presents future work.

## 2. The 3D Hermite Transform

The Hermite transform is a bio-inspired image model, it simulates some of the more relevant properties of the early vision of the human vision system (HVS): the local processing [51] and the Gaussian derivative model of the receptive fields [50,52,53]. The SHT provides a very efficient representation of oriented patterns which enables an adaptation to local orientation content at each window position over the image, indicating the direction of the two-dimensional pattern. The Hermite transform uses functions that are derivatives of Gaussians, which have wide applications in the field of computer vision and are a bio-inspired model of the human vision system. In this work, the Hermite transform serves as a theoretical framework to carry out the estimation of cardiac movement in our approach.

Gaussian windows in two dimensions have the property of being rotationally symmetric and spatially separable. Gaussian windows separated by twice the standard deviation, are a good model found for the receptive fields of perception found in psychological experiments [51]. According to the psychophysical model of HVS [52,54], through Gaussian windows, we can decompose an image into several orthogonal polynomials.

An interesting special case of 2D polynomial transforms arises when we have a window function which is separable i.e., v(x,y)=v(x)v(y)

**Figure 2:**
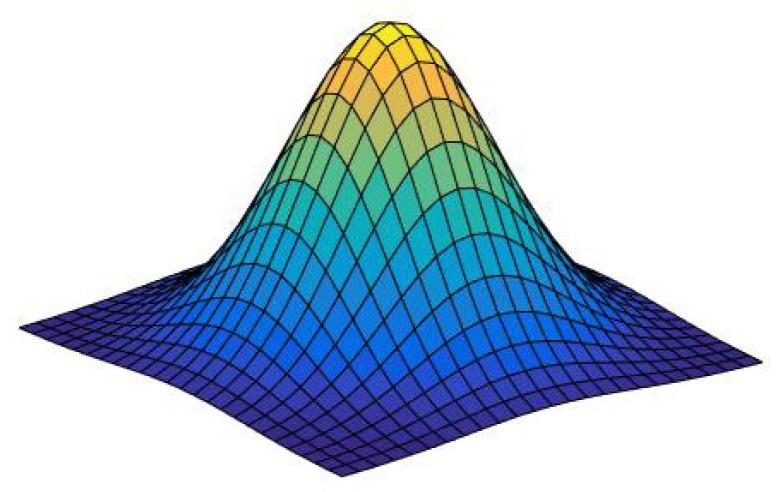
Gaussian window v(x,y).

For a perceptual standpoint and according to the scale-space theory, we will use a Gaussian window (Figure 2)

(1) $$v\left(x,y\right)=\frac{1}{\sigma \sqrt{\pi }}\exp\left(-\frac{\left(x^{2}+y^{2}\right)}{2\sigma^{2}}\right)$$

The direct Hermite transform in 3D (HT3D), is a particular case of the proposal of Martens [50,53], where a signal is localized by an analysis window and this information is expanded using polynomials orthogonal to the window. Polynomials that are orthogonal with respect to the Gaussian window function are defined by [55], so we would use the window:

(2) $$v\left(x,y,z\right)=\frac{1}{\sqrt{\sigma \sqrt{\pi }}}\exp\left(-\frac{\left(x^{2}+y^{2}+z^{2}\right)}{2\sigma^{2}}\right)$$

Physiological experiments consider using overlapping Gaussian windows separated by twice the standard deviation σ which are isotropic and that’s why we can establish that $\sigma =\sigma_{x}=\sigma_{y}=\sigma_{z}$ in accordance with the overlapping receptive fields of the human visual system [51].

The Hermite cartesian coefficients, $L_{l,m-l,n-m}$, are obtained by convolution of the original signal L(x,y,z) with the analysis filters $D_{l,m-l,n-m}\left(x,y,z\right)$ followed by subsampling on a three-dimensional mesh *S* using Equation (3):

(3) $$L_{l,m-l,n-m}\left(x_{0},y_{0},z_{0}\right)=L\left(x,y,z\right)⊗D_{l,m-l,n-m}\left(x,y,z\right),$$

where *l*, (m−l) and (n−m) denote the analysis order in *x*, *y* and *z* directions, respectively; l=0,1,⋯,m; m=0,1,⋯,n; n=0,1,⋯,N; *N* is the maximum order of the expansion that is related to the size of a cubic window of M×M×M, where N≤2*(M−1). For large values of M the discrete cubic kernel reduces to the 3D Gaussian window.

The three-dimensional Hermite filters can be represented by:

(4) $$D_{l,m-l,n-m}\left(x,y,z\right)=G_{l.m-l,n-m}\left(-x,-y,-z\right)v^{2}\left(-x,-y,-z\right)$$

wich are separable because the Gaussian window is rotationally symmetric

(5) $$D_{l,m-l,n-m}\left(x,y,z\right)=D_{l}\left(x\right)D_{m-l}\left(y\right)D_{n-m}\left(z\right)$$

and those can be computed by:

(6) $$D_{l}\left(x\right)=\frac{{\left(-1\right)}^{l}}{\sqrt{2^{l}l!}}\frac{1}{\sigma \sqrt{\pi }}H_{l}\left(\frac{x}{\sigma }\right)\exp\left(-\frac{x^{2}}{\sigma^{2}}\right)$$

$G_{l,m-l,n-m}\left(x,y,z\right)$ are a family of polynomials defined as:

(7) $$\begin{array}{c} G_{l,m-l,n-m}\left(x,y,z\right)=\frac{1}{\sqrt{2^{n}l!\left(m-l\right)!\left(n-m\right)!}}H_{l}\left(\frac{x}{\sigma }\right)H_{m-l}\left(\frac{y}{\sigma }\right)H_{n-m}\left(\frac{z}{\sigma }\right) \end{array}$$

where $H_{l}$ represents the generalized Hermite polynomials given by Rodrigues’ formula [56]

(8) $$H_{l}\left(x\right)={\left(-1\right)}^{l}\exp\left(x^{2}\right)\frac{d^{l}}{dx^{l}}\exp\left(-x^{2}\right)$$

The recovery process of the original image (inverse Hermite transform in 3D - IHT3D) consists of interpolating the Hermite coefficients through the proper synthesis filters:

(9) $$\hat{L}\left(x,y,z\right)=\sum_{n=0}^{N}\sum_{m=0}^{n}\sum_{l=0}^{m}\sum_{(x_{0},y_{0},z_{0})\in S}L_{l,m-l,n-m}\left(x_{0},y_{0},z_{0}\right)P_{l,m-l,n-m}\left(x-x_{0},y-y_{0},z-z_{0}\right)$$

where $P_{l,m-l,n-m}\left(x,y,z\right)$ can be determined by:

(10) $$P_{l,m-l,n-m}\left(x,y,z\right)=\frac{G_{l,m-l,n-m}\left(x,y,z\right)v^{2}\left(x,y,z\right)}{\sum_{(x_{0},y_{0},z_{0})\in S}v^{2}\left(x-x_{0},y-y_{0},z-z_{0}\right)}$$

for l=0,...,m; m=0,...,n and n=0,...,N.

From Equation (9), instead of to recover the original volume we obtain an approximation of the original signal $\hat{L}\left(x,y,z\right)$, where the quality of this reconstruction improves by increasing the maximum order of the expansion *N*, i.e., the size of the cubic window M [50]. In terms of the artifacts in the approximated volume $\hat{L}\left(x,y,z\right)$, small values of the cubic windows causes “speckles”, while high values result in Gibbs-phenomenon-like artifacts such as ringing and blur [57].

Thus, to determined the maximum order or the expansion *N* and in consequence the size of the cubic window M, in [57] van Dijk and Martens determined that using an expansion of the Hermite transform equal to 3, the reconstructed 2D image will contain the most quantity of AC energy (84%) according to Parseval’s theorem. In general, with N≥3 we can obtain a good reconstruction and with much greater values we will obtain a perfect reconstruction of the image.

### 3D Steered Hermite Transform

The Steered Hermite transform (SHT) is a variant of the HT that adapts to the local orientation of the image [57], it uses rotated filters which are represented as a linear combination of basis filters [58]. The orientation property of these steered Hermite filters is due to the symmetric-radial form of the Gaussian window, thus they can saw as the response of directional derivatives of the Gaussian function.

On the other hand, the SHT describes local 1D patterns in images into a smaller number of coefficients that represent the profile of the pattern perpendicular to its orientation [57].

**Figure 3:**
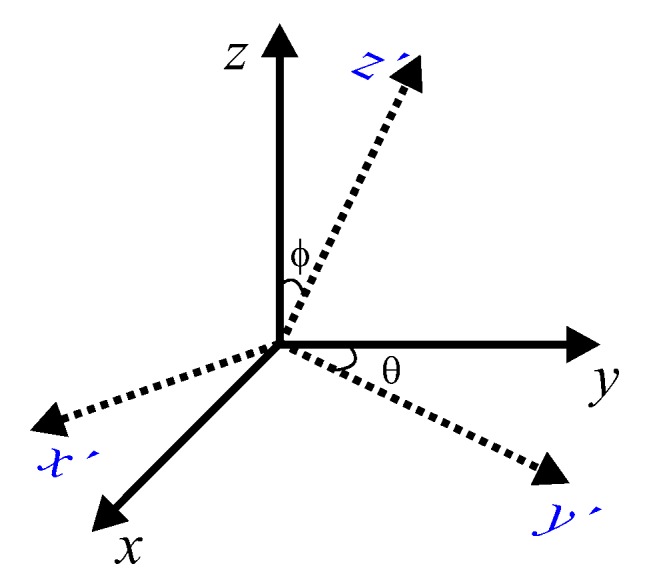
Cartesian coordinates (continuous line), steered coordinates (dotted line) and the angles θ and ϕ.

By projecting the 3D Cartesian Hermite coefficients towards the local orientation angles θ and ϕ (Figure 3), we obtain the Steered Hermite transform in 3D (SHT3D) as shown in Equation (11):

(11) $$l_{l,m-l,n-m,\theta ,\phi }\left(x_{0},y_{0},z_{0}\right)=\sum_{m=0}^{n}\sum_{l=0}^{m}\left(L_{l,m-l,n-m}\left(x_{0},y_{0},z_{0}\right)\cdot g_{l,m-l}\left(\theta \right)\cdot g_{m,n-m}\left(\phi \right)\right)$$

where $l_{l,m-l,n-m,\theta ,\phi }\left(x_{0},y_{0},z_{0}\right)$ are the 3D steered Hermite coefficients. And

(12) $$g_{j,k-j}\left(\varphi \right)=\sqrt{\left(\begin{array}{c} k\\ j \end{array}\right)}\left(\cos^{j}\left(\varphi \right)\sin^{k-j}\left(\varphi \right)\right)$$

is the cartesian angular function that expresses the directional selectivity of the filter.

To calculate the direction of maximum energy we used the coefficients from Equation (3) and the phase of the gradient given by Equations (13) and (14):

(13) $$\begin{array}{c} \theta =\arctan\left(\frac{L_{010}}{L_{100}}\right) \end{array}$$

(14) $$\begin{array}{c} \phi =\arctan\left(\frac{\sqrt{{\left(L_{100}\right)}^{2}+{\left(L_{010}\right)}^{2}}}{L_{001}}\right) \end{array}$$

where $\left[L_{1,0,0},L_{0,1,0},L_{0,0,1}\right]{\left(x,y,z\right)}^{⊤}$ are a good approximation of the 3D gradient through the Cartesian Hermite coefficients.

**Figure 4:**
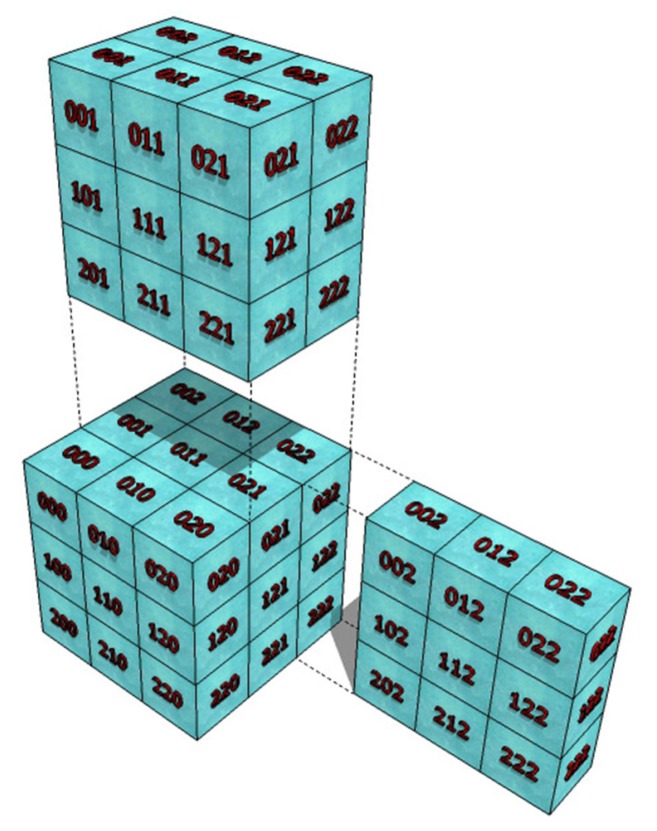
Distribution of the index of Cartesian Hermite coefficients of a second-order voxel.

In order to graphically represent the indexes of the 3D Cartesian Hermite coefficients, Figure 4 shows the distribution of order two (N=2) in each direction, in this case 27 coefficients are obtained for each voxel of a volume.

**Figure 5:**
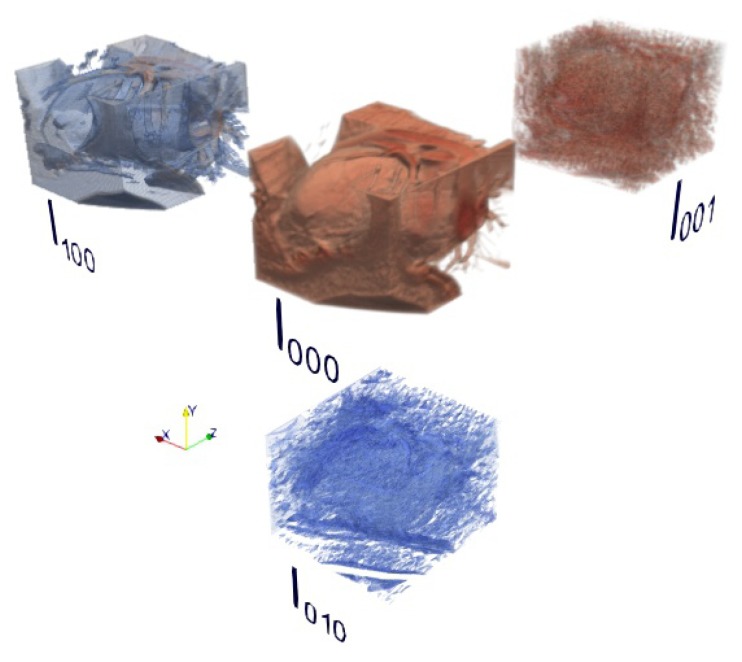
Ensemble of some Steered Hermite coefficients of a cardiac CT volume.

Figure 5 shows an example of some 3D Steered Hermite coefficients for the left ventricle of a cardiac CT volume, according to Equation (11), where we can see the steered coefficients $l_{000}$, $l_{100}$, $l_{010}$ and $l_{001}$.

## 3. Optical Flow using the Hermite Transform

One of the main disadvantages of the classic method of Horn and Schunck [11] is its low accuracy, and because of this, we use a modified version of the method proposed by Sun et al. [20], that solves such obstacle using a multiresolution approach to estimate large displacements. In addition, this modified version is combined using the Hermite Transform with the advantage that it is based on a visual biological model of the images. Consequently, the local constraints of Horn and Schunck are defined using the zero order Hermite coefficient, and the Steered Hermite coefficients are used as high order local descriptors of the visual characteristics of the volumes.

### Model

Our approach is based on the multiresolution Horn and Schunck approach reported by Sun et al. [20], it uses the SHT3D to expand the constant intensity constraint and adds the Steered Hermite coefficients constraint as shown in Equation (15):

(15) $$\left[L_{0}\left(x+w\right)-L_{0}\left(x\right)\right]+\gamma \left[\sum_{n=1}^{N}l_{n,\theta ,\phi }\left(x+w\right)-\sum_{n=1}^{N}l_{n,\theta ,\phi }\left(x\right)\right]=0$$

where $L\left(x\right)$ is a volume sequence, with $x={\left(x,y,z,t\right)}^{T}$ representing the voxel location within a domain *V*; $w:={\left(u,v,w,1\right)}^{T}$ is a vector that defines the displacement *u*, *v* and *w* of each voxel at position $\left(x,y,z\right)$ from time *t* to time $\left(t+1\right)$ in the directions *x*, *y* and *z* respectively; and γ is a weight parameter that controls the contribution of the high order descriptors. Using the HT optical flow restriction of Equation (15) we defined an energy functional that includes a smooth term to overcome the aperture problem [11] as follows:

(16) $$\begin{array}{c} \begin{array}{cc} E= & \int_{V}\left({\left[L_{0}{\left(x\right)}_{0}-L_{0}{\left(x+w+dw\right)}_{1}\right]}^{2}+\right.\\ \; & \gamma {\left[\sum_{n=1}^{N}\left\{l_{n,\theta ,\phi }{\left(x\right)}_{0}-l_{n,\theta ,\phi }{\left(x+w+dw\right)}_{1}\right\}\right]}^{2}+\\ \; & \left.\alpha |\nabla \left(w+dw\right){|}^{2}\right)dx \end{array} \end{array}$$

where α is the weight value of the smoothness term that can get information from neighbors in regions where the intensity gradient is zero (uniform regions of flow).

To simplify the notation $L_{000}\left(x\right)=L_{0}\left(x\right)$, $L_{*}{\left(x\right)}_{*}$ is the Cartesian Hermite coefficient * at time *t* and $L_{*}{\left(x\right)}_{1}$ is the Cartesian Hermite coefficient * at time t+1.

Considering linear displacements, the constant intensity term of Equation (15) can be expanded by a Taylor series as shown:

(17) $$\begin{array}{c} \begin{array}{cc} L_{0}{\left(x\right)}_{0}-L_{0}{\left(x+w+dw\right)}_{1}\approx & L_{0}{\left(x\right)}_{0}-L_{0}{\left(x+w\right)}_{1}-\\ \; & du\frac{\partial L_{0}{\left(x+w\right)}_{1}}{\partial x}-dv\frac{\partial L_{0}{\left(x+w\right)}_{1}}{\partial y}-dw\frac{\partial L_{0}{\left(x+w\right)}_{1}}{\partial z} \end{array} \end{array}$$

A particular 1D cartesian Hermite coefficient can be obtained with the inner product between the signal located by the Gaussian window and the corresponding Hermite polynomial as follows [29]:

(18) $$L_{k}=\langle L\left(x\right),H_{k}\left(\frac{x}{\sigma }\right)\rangle$$

Therefore, the spatial derivatives of the Hermite coefficients can be expressed as:

(19) $$L_{k}=\frac{\partial^{k}L\left(x\right)}{\partial^{k}x}$$

for example, we can get the following simplified derivatives for *x*:

(20) $$L_{100}\left(x+w\right)=L_{100}{\left(x\right)}_{w}=\frac{\partial L_{000}{\left(x+w\right)}_{1}}{\partial x}$$

(21) $$l_{n,\theta ,\phi ,(m+1)}{\left(x\right)}_{w}=\frac{\partial l_{n,\theta ,\phi }{\left(x+w\right)}_{1}}{\partial x}$$

also, we can define the temporal differences as:

(22) $$L_{0}{\left(x\right)}_{t}=L_{0}{\left(x+w\right)}_{1}-L_{0}{\left(x\right)}_{0}$$

(23) $$l_{n,\theta ,\phi }{\left(x\right)}_{t}=l_{n,\theta ,\phi }{\left(x\right)}_{1}-l_{n,\theta ,\phi }{\left(x+w\right)}_{0}$$

then (17) can be written as

(24) $$L_{0}{\left(x\right)}_{0}-L_{0}{\left(x+w+dw\right)}_{1}\approx -\left[L_{0}{\left(x\right)}_{t}+du\;L_{100}{\left(x\right)}_{w}+dv\;L_{010}{\left(x\right)}_{w}+dw\;L_{001}{\left(x\right)}_{w}\right]$$

Finally, we can redefine the 3D Horn-Hermite optical flow (HOF3D) functional from Equation (16) as:

(25) $$\begin{array}{cc} E\left(w\right) & =\int_{V}\left(-{\left[L_{0}{\left(x\right)}_{t}+du\;L_{100}{\left(x\right)}_{w}+dv\;L_{010}{\left(x\right)}_{w}+dw\;L_{001}{\left(x\right)}_{w}\right]}^{2}\right.\\ \; & -\gamma \sum_{n=1}^{N}{\left[l_{n,\theta ,\phi }{\left(x\right)}_{t}+du\;l_{n,\theta ,\phi ,(m+1)}{\left(x\right)}_{w}+dv\;l_{n,\theta ,\phi ,(n+1)}{\left(x\right)}_{w}+dw\;l_{n,\theta ,\phi ,(l+1)}{\left(x\right)}_{w}\right]}^{2}\\ \; & +\left.\alpha {\left|\nabla \left(w+dw\right)\right|}^{2}\right)dx \end{array}$$

Minimizing $E\left(w\right)$ with respect to *u*, *v* and *w* we obtain the following equation system:

(26) $$\begin{array}{cc} \; & -2\left(L_{0}{\left(x\right)}_{t}+du\;L_{100}{\left(x\right)}_{w}+dv\;L_{010}{\left(x\right)}_{w}+dw\;L_{001}{\left(x\right)}_{w}\right)L_{100}{\left(x\right)}_{w}\\ \; & -2\gamma \sum_{n=1}^{N}\left(l_{n,\theta ,\phi }{\left(x\right)}_{t}+du\;l_{n,\theta ,\phi ,(m+1)}{\left(x\right)}_{w}+dv\;l_{n,\theta ,\phi ,(n+1)}{\left(x\right)}_{w}+dw\;l_{n,\theta ,\phi ,(l+1)}{\left(x\right)}_{w}\right)l_{n,\theta ,\phi ,(m+1)}{\left(x\right)}_{w}\\ \; & +2\alpha |\nabla \left(u+du\right)|=0 \end{array}$$

(27) $$\begin{array}{cc} \; & -2\left(L_{0}{\left(x\right)}_{t}+du\;L_{100}{\left(x\right)}_{w}+dv\;L_{010}{\left(x\right)}_{w}+dw\;L_{001}{\left(x\right)}_{w}\right)L_{010}{\left(x\right)}_{w}\\ \; & -2\gamma \sum_{n=1}^{N}\left(l_{n,\theta ,\phi }{\left(x\right)}_{t}+du\;l_{n,\theta ,\phi ,(m+1)}{\left(x\right)}_{w}+dv\;l_{n,\theta ,\phi ,(n+1)}{\left(x\right)}_{w}+dw\;l_{n,\theta ,\phi ,(l+1)}{\left(x\right)}_{w}\right)l_{n,\theta ,\phi ,(n+1)}{\left(x\right)}_{w}\\ \; & +2\alpha |\nabla \left(v+dv\right)|=0 \end{array}$$

(28) $$\begin{array}{cc} \; & -2\left(L_{0}{\left(x\right)}_{t}+du\;L_{100}{\left(x\right)}_{w}+dv\;L_{010}{\left(x\right)}_{w}+dw\;L_{001}{\left(x\right)}_{w}\right)L_{001}{\left(x\right)}_{w}\\ \; & -2\gamma \sum_{n=1}^{N}\left(l_{n,\theta ,\phi }{\left(x\right)}_{t}+du\;l_{n,\theta ,\phi ,(m+1)}{\left(x\right)}_{w}+dv\;l_{n,\theta ,\phi ,(n+1)}{\left(x\right)}_{w}+dw\;l_{n,\theta ,\phi ,(l+1)}{\left(x\right)}_{w}\right)l_{n,\theta ,\phi ,(l+1)}{\left(x\right)}_{w}\\ \; & +2\alpha |\nabla \left(w+dw\right)|=0 \end{array}$$

Rewriting the equation system of (26), (27) and (28) in matrix form we get:

(29) $$\left[\begin{array}{ccc} A_{1} & A_{2} & A_{3}\\ A_{4} & A_{5} & A_{6}\\ A_{7} & A_{8} & A_{9} \end{array}\right]\left[\begin{array}{c} du\\ dv\\ dw \end{array}\right]=\left[\begin{array}{c} b_{1}\\ b_{2}\\ b_{3} \end{array}\right]$$

where

$A_{1}=L_{100}^{2}{\left(x\right)}_{w}+\gamma \sum_{n=1}^{N}l_{n,\theta ,\phi ,(m+1)}^{2}{\left(x\right)}_{w}$

$A_{2}=L_{100}{\left(x\right)}_{w}L_{010}{\left(x\right)}_{w}+\gamma \sum_{n=1}^{N}l_{n,\theta ,\phi ,(m+1)}{\left(x\right)}_{w}\cdot l_{n,\theta ,\phi ,(n+1)}{\left(x\right)}_{w}$

$A_{3}=L_{100}{\left(x\right)}_{w}L_{001}{\left(x\right)}_{w}+\gamma \sum_{n=1}^{N}l_{n,\theta ,\phi ,(n+1)}{\left(x\right)}_{w}\cdot l_{n,\theta ,\phi ,(l+1)}{\left(x\right)}_{w}$

$b_{1}=L_{0}{\left(x\right)}_{t}L_{100}{\left(x\right)}_{w}+\gamma \sum_{n=1}^{N}l_{n,\theta ,\phi }{\left(x\right)}_{t}\cdot l_{n,\theta ,\phi ,(m+1)}{\left(x\right)}_{w}-\alpha |\nabla \left(u+du\right)|$

$A_{4}=L_{010}{\left(x\right)}_{w}L_{100}{\left(x\right)}_{w}+\gamma \sum_{n=1}^{N}l_{n,\theta ,\phi ,(n+1)}{\left(x\right)}_{w}\cdot l_{n,\theta ,\phi ,(m+1)}{\left(x\right)}_{w}$

$A_{5}=L_{010}^{2}{\left(x\right)}_{w}+\gamma \sum_{n=1}^{N}l_{n,\theta ,\phi ,(n+1)}^{2}{\left(x\right)}_{w}$

$A_{6}=L_{010}{\left(x\right)}_{w}L_{001}{\left(x\right)}_{w}+\gamma \sum_{n=1}^{N}l_{n,\theta ,\phi ,(m+1)}{\left(x\right)}_{w}\cdot l_{n,\theta ,\phi ,(l+1)}{\left(x\right)}_{w}$

$b_{2}=L_{0}{\left(x\right)}_{t}L_{010}{\left(x\right)}_{w}+\gamma \sum_{n=1}^{N}l_{n,\theta ,\phi }{\left(x\right)}_{t}\cdot l_{n,\theta ,\phi ,(n+1)}{\left(x\right)}_{w}-\alpha |\nabla \left(v+dv\right)|$

$A_{7}=L_{001}{\left(x\right)}_{w}L_{100}{\left(x\right)}_{w}+\gamma \sum_{n=1}^{N}l_{n,\theta ,\phi ,(l+1)}{\left(x\right)}_{w}\cdot l_{n,\theta ,\phi ,(m+1)}{\left(x\right)}_{w}$

$A_{8}=L_{001}{\left(x\right)}_{w}L_{010}{\left(x\right)}_{w}+\gamma \sum_{n=1}^{N}l_{n,\theta ,\phi ,(l+1)}{\left(x\right)}_{w}\cdot l_{n,\theta ,\phi ,(n+1)}{\left(x\right)}_{w}$

$A_{9}=L_{001}^{2}{\left(x\right)}_{w}+\gamma \sum_{n=1}^{N}l_{n,\theta ,\phi ,(l+1)}^{2}{\left(x\right)}_{w}$

$b_{3}=L_{0}{\left(x\right)}_{t}L_{001}{\left(x\right)}_{w}+\gamma \sum_{n=1}^{N}l_{n,\theta ,\phi }{\left(x\right)}_{t}\cdot l_{n,\theta ,\phi ,(l+1)}{\left(x\right)}_{w}-\alpha |\nabla \left(w+dw\right)|$

Finally, in each lower-resolution level, the increment dw is estimated and, w is updated in the next high-resolution level.

In this work, we take advantage of the characteristics of the Hermite multiresolution transform, which makes it possible to improve spatial frequency locations and facilitate the analysis of local orientations at different scales [59,60]. Likewise, the HOF3D functional of Equation (25) can calculate small displacements du, dv, dw and propagate the solution to higher resolution levels. For each resolution level, an iterative method for solving linear equations was carried out.

## 4. Materials and Overview of the Method

### 4.1. Dataset Description

**Figure 6:**
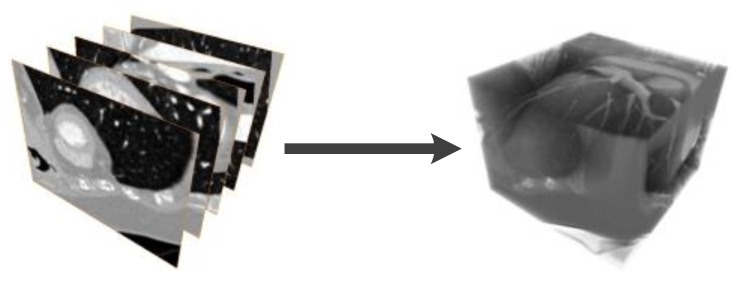
Cardiac CT images, slices and volume.

The dataset used in this work consists of two cardiac computed tomography studies (3D+t). The CT volumes were obtained in a 16-slice tomograph (at 120 kVp @ 900 mA) built with 128 detectors. The dimensions of each volume are 512×512×10 at 12 bits per pixel. The clinical protocol starts by injected a contrast agent to the patient and the study is carried out in synchrony with the electrocardiogram (ECG) signal. A cardiac CT volume used is shown in Figure 6.

It should be noted that the acquisition of cardiac images are performed in connection with the electrocardiogram and are acquired with the patient in respiratory apnea to avoid artifice by movement.

### 4.2. Ethical Approval

The Research Committee of Engineering Faculty of Universidad Nacional Autonóma de México approved this research protocol. This study was conducted in accordance with the Declaration of Helsinki.

### 4.3. Overview of the Method

**Figure 7:**
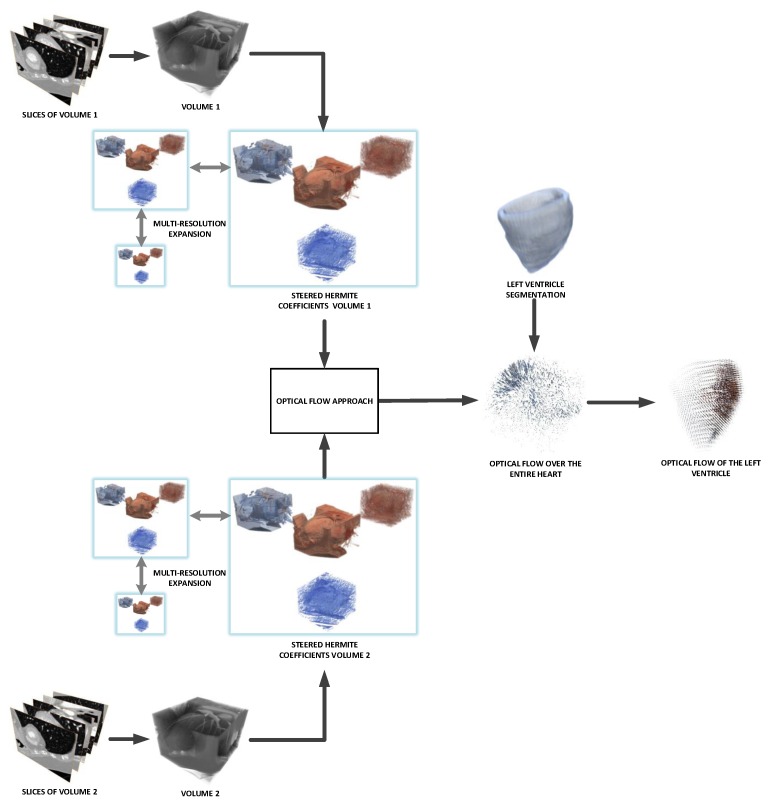
Procedure to implement the HOF3D approach.

In Figure 7 we show an overview of our proposal. First, we have the cardiac volume slices, belonging to two consecutive steps of the cardiac cycle considered, which make up volume 1 and volume 2 respectively; for each volume, we obtain a multiresolution expansion related to the coefficients of the steered Hermite transform. Such coefficients are used to carry out the calculation of the optical flow within the mentioned HOF3D approach. Once the vector field belonging to the optical flow over the whole cardiac volume was obtained, we used the portion of the volume related to the segmented left ventricle to finally obtain only the masked vectors with this part of the cardiac volume.

## 5. Experiments and Results

This section presents the results of the estimation of the optical flow with the proposed method. In our previous work [49], we use some synthetic volumes to check the expected results, in this work, dozens of cardiac volumes corresponding to medical CT images, as well as their respective segmentation of the left ventricle, were used.

The section is divided into two stages, a validation stage and a stage of 3D optical flow results of the left ventricle. In the first stage, we performed a validation of our approach, where the optimal parameters both in the Hermite transform and the 3D optical flow proposal were determined. Then, the optical flow results in 2D were compared with a set of images ground-truth and a pair of algorithms of optical flow. The optical flow results in 3D were compared with the modified and multiresolution method of Horn and Schunck [20]. Next, an analysis of robustness to noise was performed. In the second stage, the 3D optical flow results in the left ventricle, which was previously segmented, are shown and the corresponding errors of interpolation are evaluated.

The results obtained on a PC Intel(R) Core(TM) i7-4710HQ CPU running at 2.50 GHz with 16 GB of RAM have an algorithm time-consuming of 4.8 h on 4 cores, nevertheless, this can be reduced to an average of 4.5 min with parallel computing and additional cores. The optical flow in our method has good scalability, close to linear speedup, which allows us to significantly reduce processing time. The results concerning processing time are consistent with those reported in [61]. They tested two differential algorithms, Lucas-Kanade and Horn-Schunck in 3D+t, as we have also done.

### 5.1. Validation

In absence of a 3D motion ground-truth in CT images, which is used to evaluate the accuracy of the optical flow estimation, we validate our proposal in two different ways, first, by calculating a forward reconstruction using the volume $L\left(x,t\right)$ at time *t* and the 3D optical flow obtained and second, comparing our 2D approach with other methods and using a 2D dataset with known ground-truth. In both cases, we used the interpolation error, which is defined as the root mean-square (RMS) difference between the known volume $L\left(x,t+1\right)$ at time t+1 and the reconstructed volume $L_{GT}\left(x,t+1\right)$, is calculated [6,19] as we showed in the Equation (30):

(30) $$IE_{3D}={\left[\frac{1}{M}\sum_{x}{\left(L\left(x,t+1\right)-L_{GT}\left(x,t+1\right)\right)}^{2}\right]}^{\frac{1}{2}}$$

where *M* is the number of voxels.

We also computed a second measure of interpolation performance, the normalized interpolation error between an interpolated volume L(x,t+1) and a ground-truth volume $L_{GT}\left(x,t+1\right)$, which is given as in [62]:

(31) $$NE_{3D}={\left[\frac{1}{M}\sum_{x}\frac{{\left(L\left(x,t+1\right)-L_{GT}\left(x,t+1\right)\right)}^{2}}{{∥\nabla L_{GT}\left(x,t+1\right)∥}^{2}+\varepsilon }\right]}^{\frac{1}{2}}$$

that represents a gradient-normalized RMS error, where ε is a scaling constant (e.g., ε=1).

The interpolation errors are useful to know how good the calculation of the optical flow is when there is no available ground truth flow, the normalized interpolation error has the additional advantage of being normalized with respect to the magnitudes of the intensity changes that the volume of the reference.

#### 5.1.1. Hermite Transform Parameter Tuning

Although the constants, values and weight parameters are difficult to select, in Section 2, we present which are the suitable values to the cubic window and in consequence, the maximum expansion order *N* of the Hermite transform, thus, experimentally found that we achieved a good estimation of optical flow results ($NE_{3D}<0.1$) and avoiding blur artifacts for our dataset with: a cubic window of 5×5×5 pixels, i.e., a maximum expansion order of N=4 for the SHT and 5 levels of multiresolution decomposition for the SHT. Below these values, we would obtain errors 2.5 to 3 times larger than those reported. It should be noted that this strategy allows us to handle large displacements, which occur from one step to another in a cardiac cycle. On the other hand, the number of iterations greater than 50 is the one that gives us the required numerical convergence according to our tests.

#### 5.1.2. Optical Flow Parameter Sensitivity Analysis

As a first experiment, we perform a parameter sensitivity analysis to find the best values. Weight parameter γ of the HOF3D functional Equation (16) is used to weigh the contribution of the high order Hermite coefficients in those regions where the intensity does not remain constant from one volume to another. On the other hand, the softness parameter α can help recover the motion information from their neighbors in those regions where the gradient is zero, e.g., intensity homogeneous regions. It is carried out through averages from structures with high frequencies, e.g., edges and textures. Large values of α give us a smoother flow but this is relatively less important at locations with high image gradients than elsewhere.

For determining the values of the smoothness weight α and the weight parameter γ, first we compute the 3D optical flow over the cardiac CT sequences and then we analyze the behavior of the Interpolation Error (IE) and Normalized Interpolation Error (NE) metrics.

**Figure 8:**
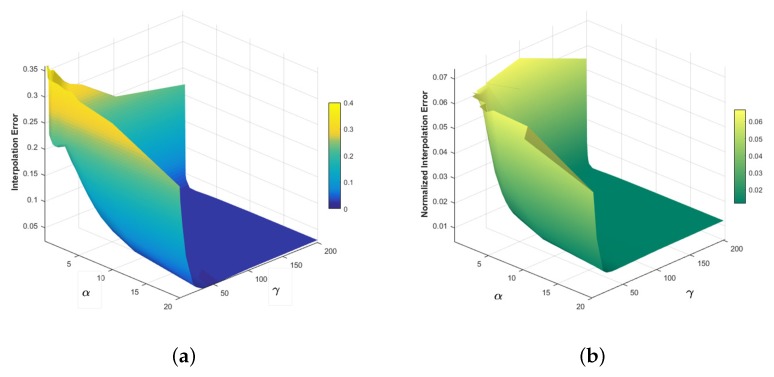
Interpolation Error (**a**) and Normalized Interpolation Error (**b**), for parameter sensitivity analysis.

From Figure 8, the curves show that the best results for IE and NE are for α≥10 and γ≥100 (bottom of the mesh).

#### 5.1.3. 2D Interpolation Errors

As we mentioned before, because we do not have a set of 3D optical flow to compare our results, as second experiment, we evaluate the performance of our 2D proposal, through a collection of well-known images. These images and their respective ground-truth optical flows can be found through [63], which still have great use and relevance today. They defined sequences with non-rigid movements where the optical flow was determined following a hidden fluorescent texture.

Table 2 shows the calculation of the interpolation error (Equation (30)) but in 2D. We choose a set of five data. HOF2D is the Horn-Hermite optical flow in 2D approach. We compare our HOF2D algorithm, along with another pair of algorithms and the ground-truth flows provided in [63]. The parameters used for HOF2D are the same as those described in Section 5.1.1 and Section 5.1.2, N=4, 5 levels of multiresolution decomposition for the SHT in 2D, α≥10 and γ≥100, except that the necessary iterations, which can range from 20, to provide the best results. The best results are highlighted in bold and, although our approach is not always the best, it is close to the best results in each case.

With the same set of data and algorithms as Table 2, Table 3 presents the calculation of the normalized interpolation error in 2D based on (Equation (31)). The best performances are highlighted in bold and most of them are in the HOF2D column. We must remember that the normalized interpolation error is is a weighted RMS average of the pixels, wich use the image gradient as a weight factor. The normalized interpolation error compensates for the difference between the interpolation errors and the flow obtained because it gives less weight to the discontinuous regions and more weight to the regions without texture.

#### 5.1.4. 3D Interpolation Errors

**Figure 9:**
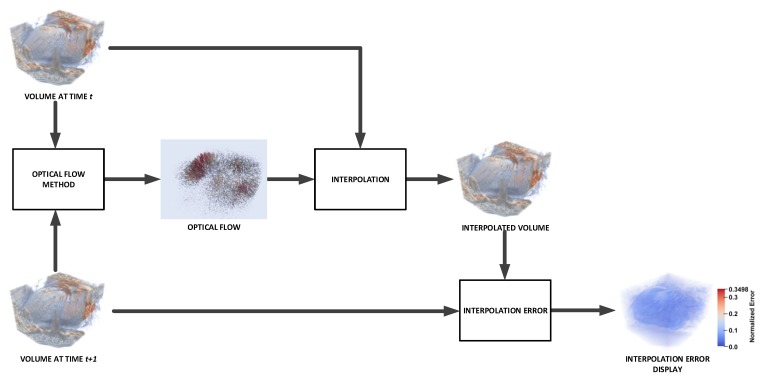
Steps to calculate and visualize the Interpolation Error.

To evaluate the accuracy of the HOF3D method, we compared it with the 3D variant of the method of Sun et al. [20] We calculated the corresponding interpolation errors (IE and NE) using both proposals. Figure 9 presents a diagram of the steps to calculate the interpolation errors.

**Figure 10:**
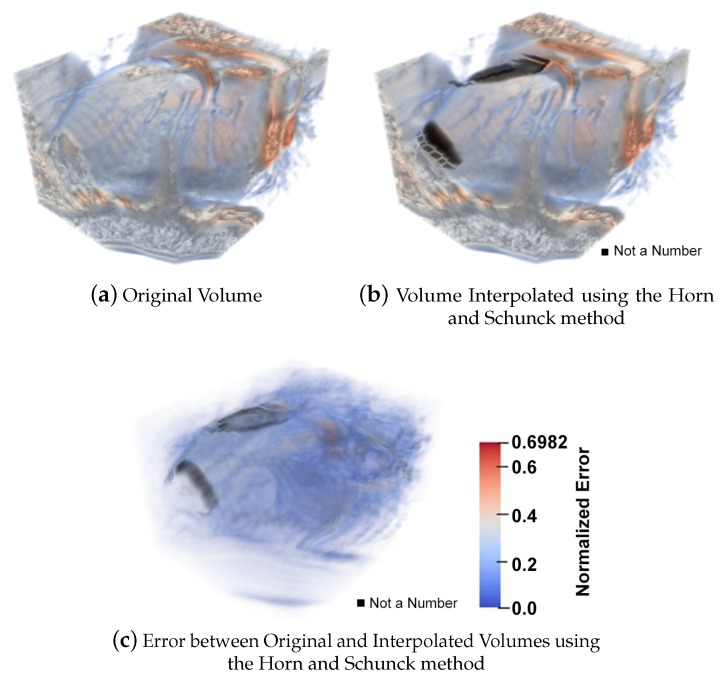
A cardiac CT volume showing the original volume, the interpolated volume using the 3D variant of the method of Sun et al. [20] and the error between the original volume and its interpolated result.

Figure 10 shows a cardiac CT volume where we can observe the original volume (Figure 10a), the interpolated volume (Figure 10b), the difference between the original volume and its interpolated result (Figure 10c) using the 3D variant of the method of Sun et al. [20]. The results were compared with a modified version of the Sun method in 3D and for different noise levels. Both algorithms used were optimized and the evaluation of the results was carried out by means of a forward reconstruction, from the volume at time *t* to time t+1, through the 3D optical flow obtained. The interpolation error display is a visualization of the terms within the summation in Equation (31).

**Figure 11:**
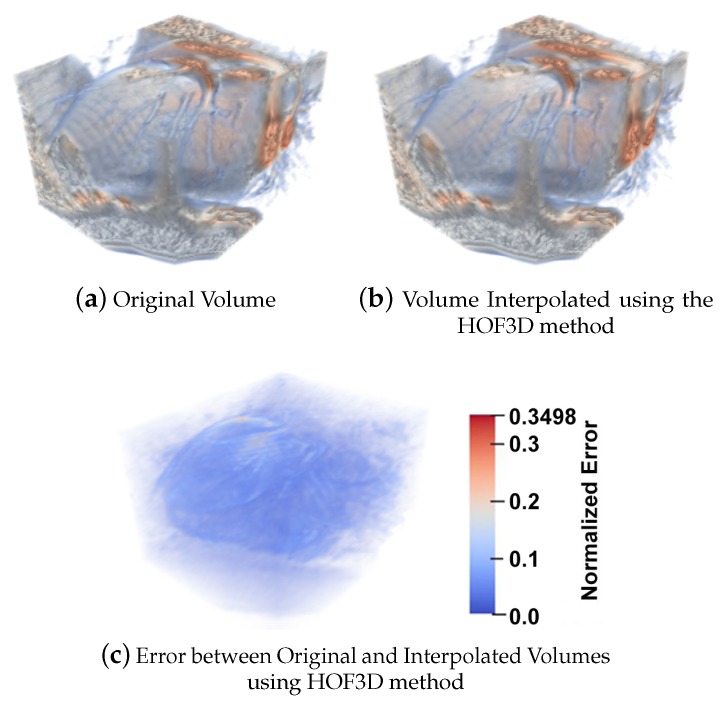
A cardiac CT volume showing the original volume, the Interpolated Volume using the HOF3D method and the error between the original volume and its interpolated result.

On the other hand, in order to compare our method, in Figure 11 we show the same cardiac CT volume as in Figure 10 where, again, we can observe the original volume (Figure 11a), the interpolated volume (Figure 11b), the difference between the original volume and its interpolated result (Figure 11c) using our HOF3D method.

**Figure 12:**
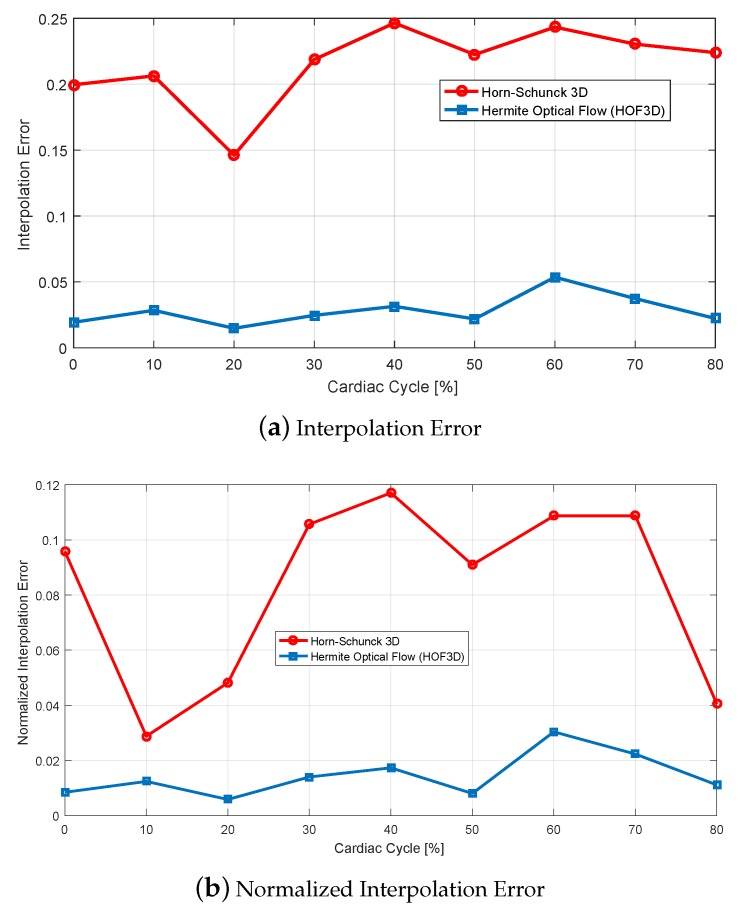
Interpolation Error and Normalized Interpolation Error. For the 3D Horn-Schunck (red dashed line) and Hermite Optical Flow in 3D (blue solid line) methods. From sequences of cardiac CT volumes.

In Figure 12, we show the interpolation errors obtained using the Sun et al. [20] and the HOF3D methods through the whole cardiac cycle (0% to 90%) for two CT sequences. In both sequences, we can observe the beginning of the increase in the interpolation error from 20% to 30%, when the contraction movement occurs and from 50% to 60% of the cardiac cycle, in full dilation movement. This is where we have a couple of cardiac movements of greater magnitude.

We can observe in the plots of Figure 12, both for the interpolation error and the normalized interpolation error, that even in each of the stages of the complete cardiac cycle, the HOF3D method gives better results and lower errors are obtained.

#### 5.1.5. Robustness to Noise

**Figure 13:**
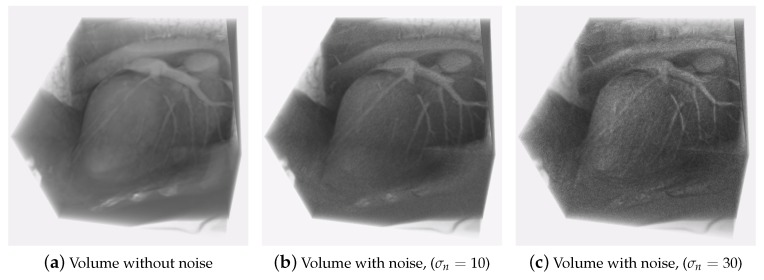
Volume with pseudo-random noise.

As final experiment, we carried out an analysis of robustness to noise of the proposed method. For this, we added Gaussian noise, with different standard deviations ($\sigma_{n}=0,5,10,15,20,30$) and zero mean, to the cardiac volumes. In Figure 13 we can see one of the volumes used for the test, with three different values of $\sigma_{n}$.

Table 4 shows the interpolation error and the normalized interpolation error for the noise levels given for the HOF3D method, using optimized parameters α=10, γ=100 and N=4. We can observe that although the standard deviation of the introduced noise grows, the interpolation error and the normalized interpolation error remain small, this is because the coefficient of order 0 of the Hermite transform $L_{000}\left(x\right)$ (Equations (3), (11) and (16)), contains a smoothed version of the original volume and this DC coefficient allows to reduce any component of high-frequency noise, additionally, in our approach, the steered Hermite coefficients use Gaussian derivatives, which incorporate information from neighboring voxels in the structure of cardiac volumes, which makes the proposed algorithm more robust to this type of noise [13,65]. By the other hand, it should be noted that although the errors are low, the addition of noise represents an increase in the interpolation error of 71.4% and for the normalized interpolation error of 80%, comparing one test without noise and the other test with noise of $\sigma_{n}=30$.

### 5.2. 3D Optical Flow Results

In this section, we show the 3D optical flow estimation computed on CT volumes for a whole cardiac cycle. For descriptive purposes, only some representative parts of such a cardiac cycle are shown. In most cases, the display of the magnitudes of the optical flows was exaggerated in order to observe the qualitative characteristics of the movements.

**Figure 14:**
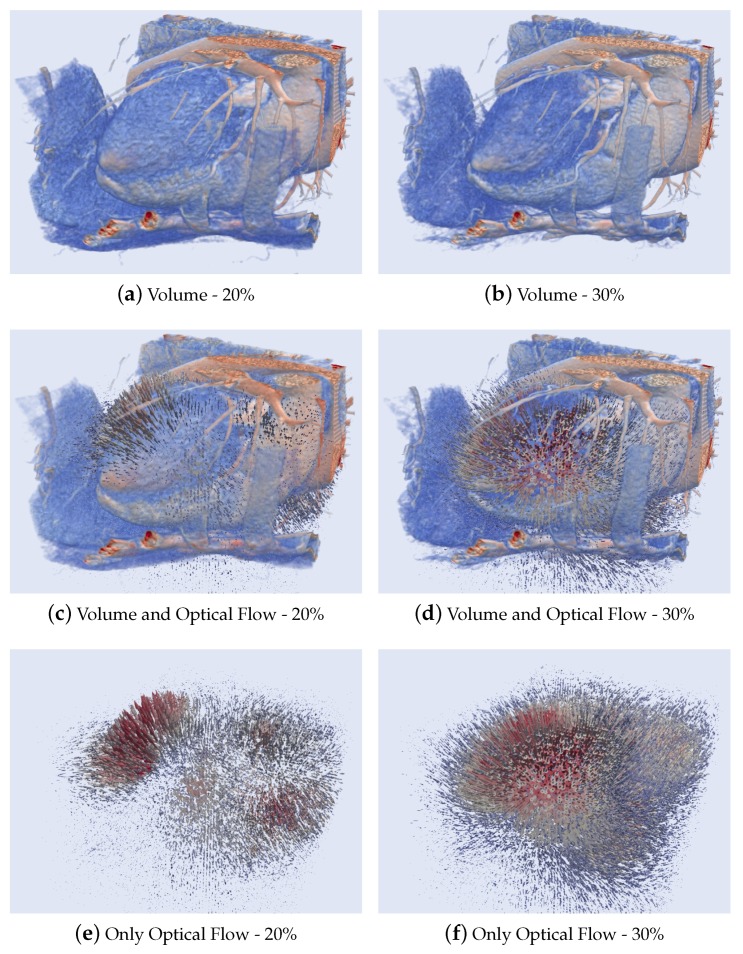
Results of 3D Optical Flow of a segmented cardiac CT volume computed at phases 20–30%.

Figure 14 shows the results of a 3D Optical flow of two cardiac CT volumes computed at phases 20–30% (when a contraction movement occurs) using the HOF3D method. Figure 14a,b show two phases of the cardiac cycle of volume (for better viewing a cut of that volume was made). Figure 14c,d show the same phases of volume along with the three-dimensional optical flow field. Finally, Figure 14e,f illustrate only the optical flow.

**Figure 15:**
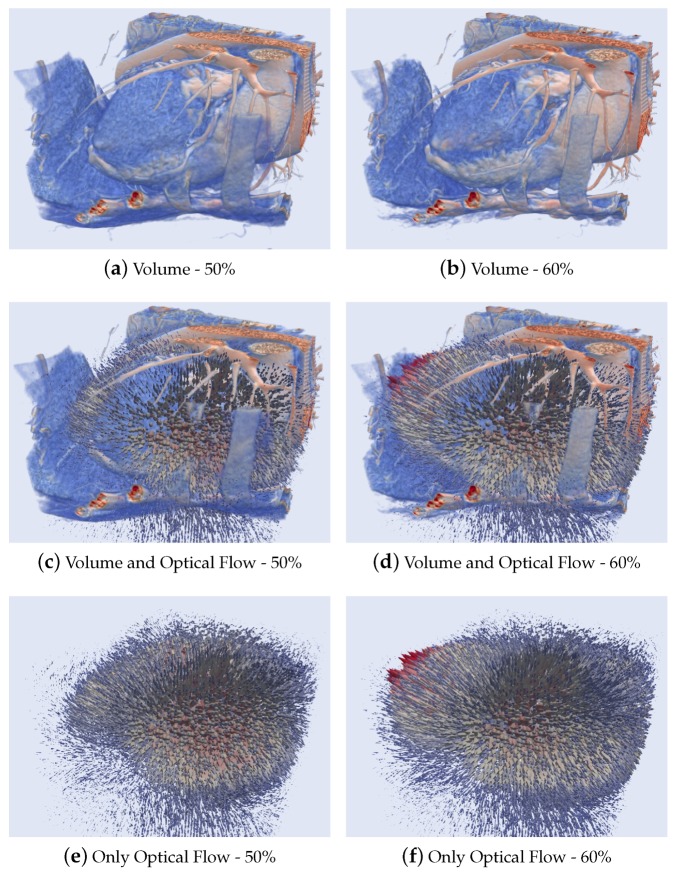
Results of 3D Optical Flow of a segmented cardiac CT volume computed at phases 50–60%.

Figure 15 presents the results of a 3D Optical flow of two cardiac CT volumes computed at phases 50–60% (when a dilation movement occurs) using the HOF3D method. Figure 15a,b show two phases of the cardiac cycle of volume (for better viewing a cut that volume was made). Figure 15c,d show the same phases of volume along with the three-dimensional optical flow field. Also, Figure 15e,f present only the optical flow.

#### 5.2.1. 3D Optical Flow Estimation of the Left Ventricle

The importance of the study of the left ventricle has been established extensively. The left ventricle adapts, for example, to arterial hypertension and this leads to the development of different geometric patterns [66]. For a better understanding of some diseases, the movement of the left ventricle has been studied during the cardiac cycle in normal subjects and patients with coronary arterial disease, mitral stenosis or atrial septal defect [67]. Works describing the global and local movement have been presented, focusing mainly on the left ventricle [68]. To present the optical flow estimation of the left ventricle, first, a segmentation of it is required. We use the level sets method of Osher and Sethian [69]. This method is a powerful, suitable and flexible approach to segmentation of CT volumes where there aren’t well-defined boundaries. The level sets method was applied to the CT volumes using the Seg3D tool [70]. For this tool, a seed volume is used to find similar regions to the original one. Then, the segmented region will be expanded to surrounding pixels that match the statistics of the original seeded area. The spread may also be retracted in some instances if the seeded areas do not match certain criteria (edge weight and threshold range). Until the convergence, the algorithm will be expanded (or contracted) to the segmented region.

**Figure 16:**
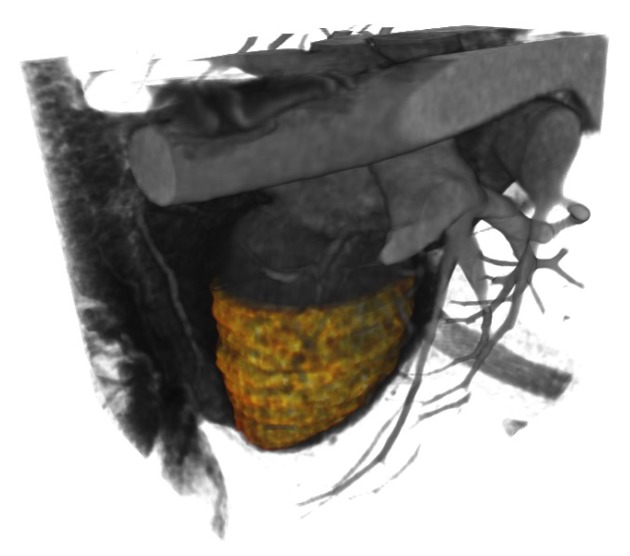
A whole cardiac volume and its left ventricle segmented.

Figure 16 shows an example of the segmentations obtained (colored region) in the context of their location within the whole cardiac volume.

**Figure 17:**
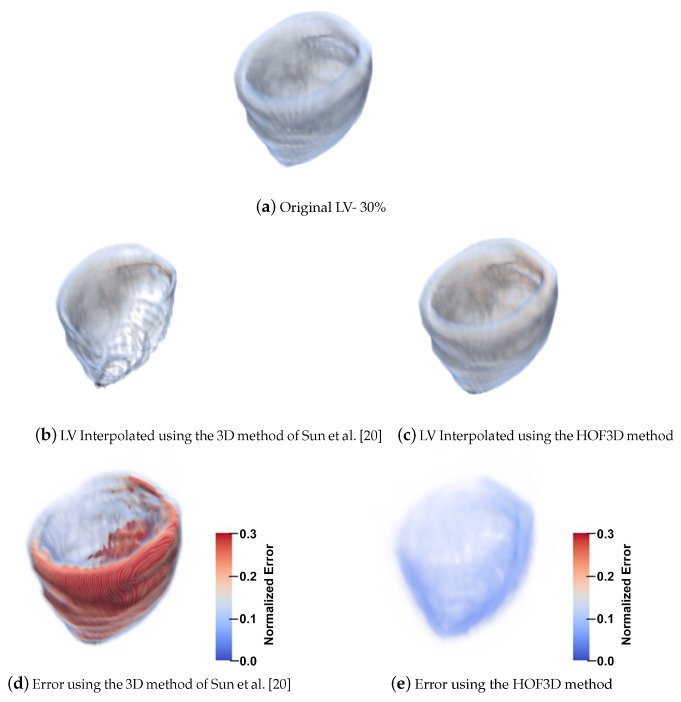
Interpolation Errors for the left ventricle at 30%.

Similarly to the work done in Section 5.1.4, and if we focus on the phase where a contraction movement occurs for the left ventricle, we can observe the interpolation error for a left ventricle segmented showing the original volume in Figure 17a, the interpolated volume using the 3D variant of the method of Sun et al. [20] are in Figure 17b, the error between the original volume and this interpolated result, in Figure 17d. Also, the optical flow is calculated by the HOF3D method. Then, the interpolation of the left ventricle is obtained, which is shown in Figure 17c. The difference between the original volume and that interpolated volume can be observed in Figure 17e.

**Figure 18:**
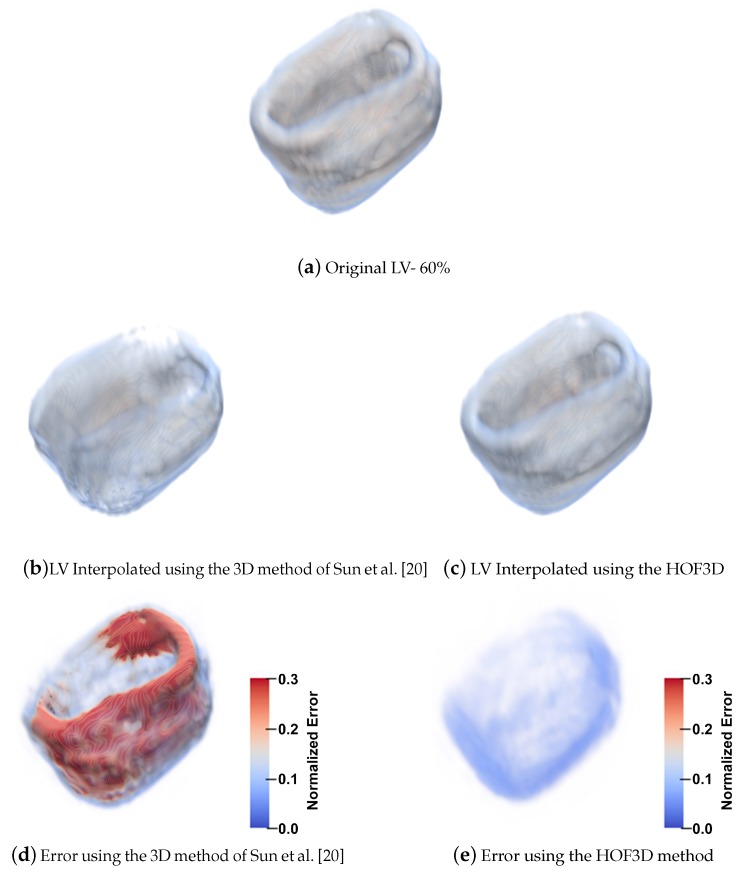
Interpolation Errors for the left ventricle at 60%.

Another example of interest, where there is more interpolation error, is the 60% of cardiac phase, it is also when a dilatation movement occurs for the left ventricle. We can observe the interpolation error for a left ventricle segmented for the original volume in Figure 18a, the interpolated volume using the 3D method of Sun et al. [20] in Figure 18b, the error between the original volume and this interpolated result in Figure 18d. Also, the optical flow is calculated by the HOF3D method. Then, the interpolation of the left ventricle is obtained and shown in Figure 18c. The difference between the original volume and that interpolated volume can be observed in Figure 18e.

**Figure 19:**
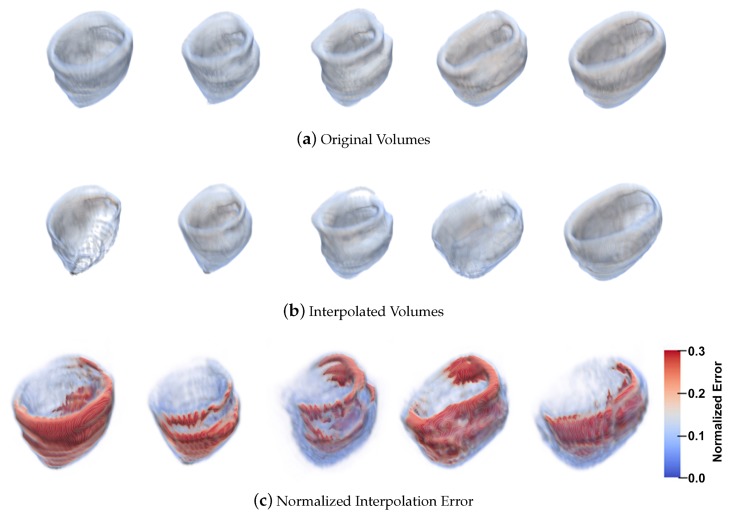
Left Ventricle from 30% to 70% of the cardiac cycle and the results with 3D method of Sun et al. [20].

Figure 19 contains the results of the normalized interpolation error with the Sun et al. [20] method. In Figure 19a we present a set of volumes from 30% to 70% of the cardiac cycle, in Figure 19b, the interpolated volumes corresponding to each stage of the cardiac cycle are shown. Figure 19c graphically displays the normalized interpolation error for the volumes of sections a and b respectively.

**Figure 20:**
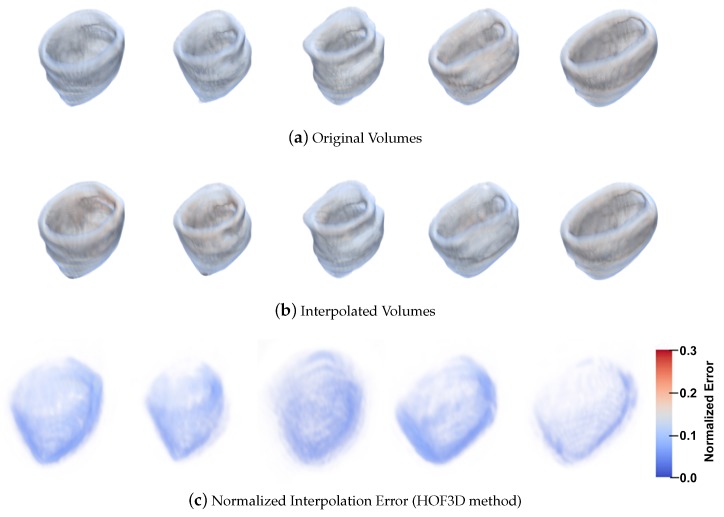
Left Ventricle from 30% to 70% of the cardiac cycle and the results with HOF3D method.

Figure 20 contains the results of the normalized interpolation error with the HOF3D method. Figure 20a presents a set of volumes from 30% to 70% of the cardiac cycle, Figure 20b shows the interpolated volumes corresponding to each stage of the cardiac cycle. Figure 20c graphically displays the normalized interpolation error for the volumes of sections a and b respectively.

**Figure 21:**
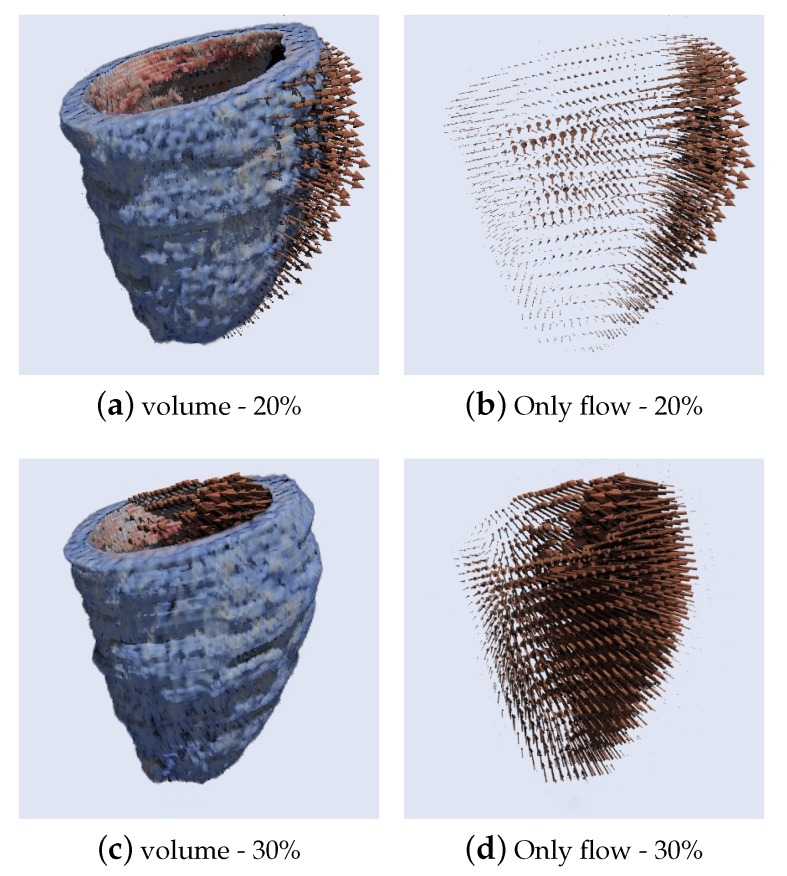
Results of 3D Optical Flow of a segmented cardiac CT volume (left ventricle) computed at phases 20–30% (contraction movement).

**Figure 22:**
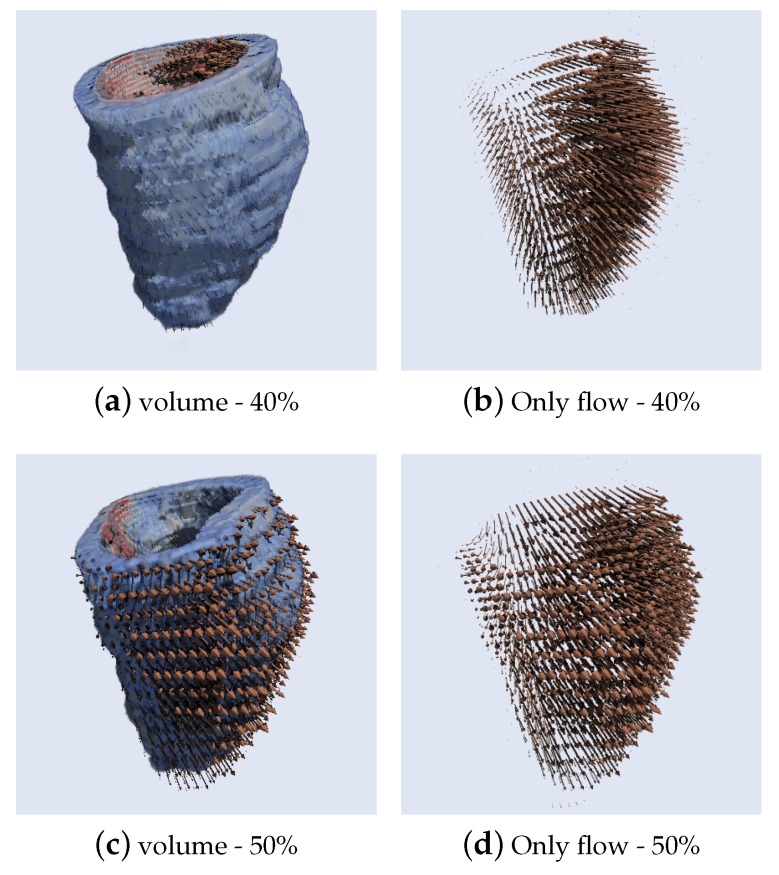
Results of 3D Optical Flow of a segmented cardiac CT volume (left ventricle) computed at phases 40–50% (relaxation movement).

Figure 21 and Figure 22 present the results of a 3D Optical flow of two segmented cardiac CT volumes, showing a contraction and relaxation movement respectively using the HOF3D method. Figure 21a,c are two phases of the segmented cardiac cycle of volume computed at phases 20–30%, the same way as Figure 22a,c but in phases 40–50%. Figure 21b,d, Figure 22b,d show only their respective optical flows.

**Figure 23:**
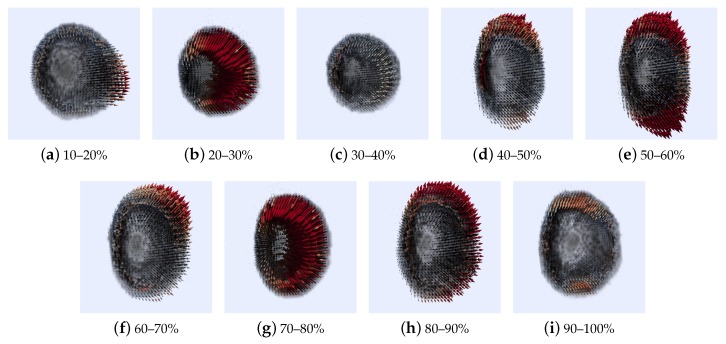
Results of 3D Optical Flow of a segmented cardiac CT volume (left ventricle–short axis).

Figure 23 contains the results corresponding to the optical flow calculated with the HOF3D method. We can observe a set of left ventricle volumes from 10% to 100% of the cardiac cycle.

## 6. Discussion

In this section we will talk about the results obtained, their interpretation and the level of relevance reached.

In the first group of results (Figure 14 and Figure 15), the entire cardiac volume and its respective calculated optical flows can be observed in context. The best way to display this 3D flow has been attempted. Despite a large number of 3D arrows of different sizes, it is possible to observe either the contraction pattern (Figure 14) or the expansion pattern (Figure 15). The optical flow is shown using of Paraview [71,72] whose style of representing the vectors of the optical flow is similar to the previous works in [73] and recently in [74].

We focused on estimating the movement in one of the most important structural parts of the heart, the left ventricle. To achieve this goal we have segmented that heart region, which is shown in Figure 16. We can see examples in [75,76] of the deployment of the three-dimensional vectors of the obtained optical flow. However, in all the remaining figures within the set of results obtained, rather than deploying the obtained optical flow vectors, we decided to show graphically, a measure of the performance achieved in the estimation of cardiac movement, specifically in the left ventricle. The relevance of the figures thus represented is that the errors obtained can be observed graphically, first in the interpolated volumes (Figure 17 and Figure 18, items b and c), where we can compare a similar and known method with the proposed one. In the same figures mentioned, the interpolation error is observed through of a three-dimensional representation that matches the analyzed volumes (Figure 17 and Figure 18, items d and e). In those figures, the biggest errors are the ones represented in red and the smallest tend to blue.

We observe a more extensive sequence of of the cardiac cycle (five phases) in order to provide greater clarity. In this selection of phases of the cardiac cycle we can observe the movement of contraction and dilatation in the left ventricle. In Figure 19 and Figure 20 in part a, we observe the original volumes. In Figure 19 and Figure 20 part b, we see the interpolated volumes with a comparison between the two methods. In Figure 19 and Figure 20 item c, we see a graphical representation of the normalized interpolation error.

Finally, Figure 21 and Figure 22 present more explicitly the segmented left ventricle and their respective optical flows during the contraction and relaxation movements. Figure 23 shows the segmented volumes from the short view concerning all phases of the cardiac cycle (from 10% to 100%). Motion vectors were exaggerated for clarity. In the field of medical images, and in addition to the cardiac movement, this approach can be used with benefit in pulmonary movement. In general, in applications where we have three-dimensional data, such as cardiac and pulmonary medical images, stereoscopic images and video, 3D meteorological data, volumes formed by point clouds in general. Where we want to characterize how they evolve over time. For future work, there is a great margin of opportunity to improve the times in the calculations of the Hermite transform, for which a faster version was not used. For the energy functional used, some other local and global characteristics can be incorporated that allow us to further reduce the uncertainties obtained.

## 7. Conclusions

In this paper, we have proposed a method to estimate the optical flow completely in 3D+t, that is, in a three-dimensional space (x,y,z) plus time, because the analysis of two-dimensional motion restricts all possible deformations in the different directions of reference (i.e., radial, circumferential and longitudinal). Therefore, the three-dimensional motion analysis can overcome such limitations by describing better all directions of deformations.

Our approximation of motion estimation has included the well-known differential method of Horn and Schunck with the additional information provided by the coefficients of the Steered Hermite transform used within the restriction terms of the function to be minimized. The Steered Hermite transform is a model that incorporates some important properties of the first stages of the human visual system, such as the overlapping Gaussian receptive fields, the Gaussian derivative model of early vision [52], and a multiresolution analysis [60,77]. This proposed algorithm is more robust to noise due to the advantage represented by the analysis of the spatial scale provided by the Hermite transform itself that can be determined for objects at different spatial dimensions. Additionally, and due to the calculation of high order Gaussian derivatives, the estimation of the movement can be improved by including structures related to them.

We evaluated the results obtained using two measurements on the interpolation errors, with these errors we also adjusted the most appropriate parameters in the different cardiac sets considered. We observed that interpolation errors increased around the phases where movements occur most rapidly (the contraction phase). We were able to verify that the proposed method (HOF3D) has lower interpolation errors compared to the modified 3D method of Sun et al. [20].

We isolated the three-dimensional flow vectors corresponding to the left ventricle, over the entire cardiac cycle. We calculated the interpolation errors obtained with our method, comparing the results with the other method already mentioned. The results were plotted graphically, showing that the largest errors were colored in red, as shown in the figures. Again, our method has minor interpolation errors.

Our proposal also aims to contribute to a better understanding of cardiac movements and, with this, to make feasible the detection of some possible diseases. We consider that because the cardiac organ is immersed in a three-dimensional space, the best way to represent its movements should be in the same three-dimensional space. Future work may focus on recognizing the cardiac movement patterns related to the vectors obtained in our three-dimensional optical flow approximation. 

## Figures and Tables

**Table 1:** 2D/3D+t optical flow estimation approaches.

Paper	OF Model (2D/3D+t)	Method	Application	Evaluation Metric
Proposed method	3D	Using the 3D Steered Hermite Transform	Left ventricle CT sequences	Interpolation errors in 3D
Ranjan et al. [34]	3D	A 3D model human body and a CNN	Estimate human flow fields	End point error
Alexiadis et al. [35]	2D	Minimizing a cost functional	3D flow estimation	Mean angular error on synthetic images
Queiros et al. [41]	3D	Anatomically affine optical flow	Left ventricle tracking	Distance and Dice metrics
Patil et al. [24]	2D	Farnebäck	Emotion recognition	Accuracy of 6 emotions
Saleh et al. [26]	2D	Lucas-Kanade	Heart Localization	Accuracy on localizing
Baghaie et al. [30]	2D	Gabor, Schmid and steerable filters	2D flow estimation	Angular and interpolation errors
Rodriguez et al. [25]	2D	Horn & Schunck	Cardiac motion estimation	Mean square error

**Table 2:** Interpolation Error Calculation.

Ground Truth Images	Ground Truth Flow	Horn-Schunck [64]	Farnebäck [64]	HOF2D
dimetrodon	2.641	8.589	3.127	**2.865**
groove2	10.439	23.492	8.831	**10.353**
groove3	19.401	32.351	**15.703**	17.460
urban3	9.870	17.727	9.489	**8.122**
venus	8.813	20.659	**5.847**	8.835

**Table 3:** Normal Interpolation Error Calculation.

Ground Truth Images	Ground Truth Flow	Horn-Schunck [64]	Farnebäck [64]	HOF2D
dimetrodon	0.207	0.546	0.382	**0.270**
groove2	0.418	0.860	0.385	**0.329**
groove3	0.990	1.622	0.626	**0.532**
urban3	2.325	2.452	1.342	**0.700**
venus	0.801	1.376	0.434	**0.348**

**Table 4:** Interpolation Error and Normalized Interpolation Error computed for a cardiac volume with several standard deviations $\sigma_{n}$ of Gaussian noise.

Gaussian Noise ($\sigma_{n}$)	Interpolation Error	Normalized Interpolation Error
0	0.03190	0.01696
5	0.03499	0.01954
10	0.03778	0.02168
15	0.04295	0.02563
20	0.04597	0.02779
30	0.05468	0.03387

## Abbreviations

The following abbreviations are used in this manuscript:

3D+t	Three-dimensional space plus time
2D+t	Two-dimensional space plus time
HT	Hermite transform
SHT	Steered Hermite transform
HVS	Human vision system
CT	Computed tomography
CVD	Cardiovascular diseases
LV	Left ventricular
MRI	Magnetic resonance imaging
HT3D	Hermite transform in 3D
IHT3D	Inverse Hermite transform in 3D
SHT3D	Steered Hermite transform in 3D
HOF3D	Horn-Hermite optical flow in 3D
ECG	Electrocardiography
IE	Interpolation error
NE	Normalized interpolation error
CNN	Convolutional Neural Network
